# The Protection of RC Columns by Bio-Inspired Honeycomb Column Thin-Walled Structure (BHTS) Under Impact Load

**DOI:** 10.3390/biomimetics9120759

**Published:** 2024-12-13

**Authors:** Shijie Wang, Hongxiang Xia, Yuncui Zong, Jianjun Liang, Ripeng Zhu

**Affiliations:** 1 College of Civil and Architectural Engineering, Heilongjiang Institute of Technology, Harbin 150050, China; 2 School of Environment and Civil Engineering, Dongguan University of Technology, Dongguan 523808, China; 3 School of Mechanical Engineering, Tianjin University, Tianjin 300354, China; 4 Heilongjiang Highway Construction Center, Harbin 150040, China; 5 Heilongjiang Construction Investment Group Co., Ltd., Harbin 150046, China

**Keywords:** bio-inspired honeycomb structure, reinforced concrete, structural impact, energy absorption, numerical simulation

## Abstract

The bio-inspired honeycomb column thin-walled structure (BHTS) is inspired by the biological structure of beetle elytra and designed as a lightweight buffer interlayer to prevent damage to the reinforced concrete bridge pier (RCBP) under the overload impact from vehicle impact. According to the prototype structure of the pier, a batch of scale models with a scaling factor of 1:10 was produced. The BHTS buffer interlayer was installed on the reinforced concrete (RC) column specimen to carry out the steel ball impact test. Then, the modified numerical model was subjected to the low-energy input impact test of the steel ball without energy loss during the falling process at the equivalent height of 1.0–3.5 m, and the dynamic response characteristics of the RC column were analyzed. By comparing the impact force and impact duration, maximum displacement, and residual displacement in the impact model, the BHTS buffer interlayer’s protective effect on RC columns under lower energy lateral impact was evaluated. Later, a high-energy input lateral impact test of a steel ball falling at an equivalent height of 20.0 m was carried out. According to the material damage, dynamic response, and energy absorption characteristics in the impact model, the failure process of the RC columns was analyzed. The results showed that BHTS absorbed 82.33% of the impact kinetic energy and reduced 77.27% of the impact force, 86.51% of the inertia force, and 64.86% of the base shear force under the failure mode of a 20 m impact condition. It can transform the shear failure of the RC column into bending failure and play an effective protective role for the RC column. This study can provide useful references for collision prevention design in practical engineering.

## 1. Introduction

The honeycomb structure has high strength, high stiffness, and lightweight mechanical properties [1,2,3] and has been widely used in the civil engineering, transportation, and aerospace fields [4,5]. In addition, the honeycomb structure also has excellent energy absorption (EA) capacity. In recent years, some scholars have studied the EA of different types of honeycomb structures [6,7,8]. It is believed that honeycomb structures have excellent specific energy absorption (SEA) and can be used in impact resistance scenarios. The damage caused by impact load will lead to the decrease in material properties, especially compressive strength, which will lead to the catastrophic failure of materials in the whole life of the structure. Therefore, it is a suitable choice to use the honeycomb structure as the protective structure of impact load.

The development of the honeycomb structure is closely combined with bionic engineering. Extensive research has been conducted on the intricate biological formations found in certain animals and plants, showcasing their remarkable mechanical capabilities. The mechanical properties of these bionic honeycomb structures far surpass those of conventional honeycomb structures (CHS). Starting from the internal structure of beetles, Xiang and Du [9] proposed a new type of honeycomb structure, namely, the bio-inspired honeycomb column thin-walled structure (BHTS), which fills columns in different ways. Two kinds of BHTSs were designed to study the EA characteristics of both kinds of BHTS under axial impact load. The findings revealed that BHTS exhibited superior EA characteristics compared to CHS. The SEA of BHTS-2 (the second type) was 35.97% larger than that of CHS; therefore, BHTS has great application potential in EA scenarios. A. Sabah et al. [10] developed a new type of bio-inspired honeycomb sandwich beam (BHSB) based on the woodpecker head configuration for repeated low-speed impact resistance improvement. In all cases, compared with the conventional honeycomb sandwich beam (HSB), the stress of the bottom skin of the BHSB was significantly smaller, the damage area was significantly lower, and the number of impacts is greater. In addition, the impact efficiency index of BHSB was 1.65 to 16.22 times that of HSB, indicating that the repeated impact resistance of BHSB was better than that of HSB. Inspired by the microstructure of bamboo vascular bundles, Hu et al. [11] studied the EA characteristics of the bionic honeycomb tubular nested structure (BHTNS). The EA properties of BHTNS during axial crushing were thoroughly investigated through drop hammer testing, computer modeling, and theoretical examination. The results showed that the SEA performance of BHTNS is as high as 29.3 J/g, which can provide a reference for the design of high-efficiency buffer absorbers. N. San Ha et al. [12] studied the mechanical properties and EA characteristics of the proposed bio-inspired hierarchical circular honeycomb (BHCH) by combining experimental, numerical simulation, and theoretical analysis. The SEA of the proposed BHCH was 45.3% and 71.2% higher than that of the conventional circular honeycomb (CH) with a similar thickness core or volume core, respectively. This research proved that the proposed BHCH simulated tree structure showed a significant enhancement effect as an energy absorber and has great potential in various engineering applications.

In recent years, as a new type of bionic structure, BHTS has become well known by researchers for its excellent EA capacity [13,14]. Many studies have been carried out on BHTS around the world. In 2018, Hao and Du [15] studied the internal structure of elytra and proposed three bio-inspired honeycomb column thin-walled structures (BHTSs). Then, the crushing behavior and EA characteristics of BHTSs under axial impact load were studied via numerical simulation. The results showed that BHTSs have excellent SEA and have potential applications in aerospace engineering. In 2023, Xia et al. [16] studied the EA of BHTS based on the work of Hao and Du [15], considering the strain rate and material damage of aluminum alloy materials. The experimental results showed that the strain rate effect significantly improved the EA index of each BHTS in the out-of-plane compression experiment. In 2023, Wang and Xia [17] introduced BHTS into the drop hammer test impact protection of RC slabs. Their findings indicated that the proposed BHTS buffer interlayer has a highly effective protective impact on RC slabs during drop hammer testing. The BHTS buffer interlayer offers a potential solution for enhancing the EA of RC structures commonly found in defensive structural elements like floors and building walls. In 2023, Xia et al. [18] applied BHTS as a replaceable additional anti-collision structure to civil engineering. The dynamic response of the BHTS-protected simply supported beam structure was studied and explained, and the limit displacement prediction model of the BHTS-RC beam was given. The protective device can effectively convert the shear failure damage mode of RC beams into bending damage, which greatly reduces the dynamic response of RC beams. In 2024, Xia et al. [19] conducted a theoretical study on the BHTS proposed by Hao and Du [15]. The average crushing force and EA of BHTS were derived using the simplified super folding element (SSFE) theory. The results showed that the EA capacity of BHTS was much higher than that of CHS. In this regard, an anti-collision composite structure combining ultra-high performance concrete (UHPC) and BHTS (UHPC-BHTS) was proposed. After research, the structure can effectively protect the reinforced concrete bridge pier (RCBP) and can be used as an additional anti-collision structure for pier impact and structural protection scenarios. In 2024, Xia et al. [20] studied the impact damage failure mechanism and EA mechanism of the UHPC-BHTS composite structure. The results showed that the UHPC-BHTS composite structure can significantly absorb the impact of kinetic energy, greatly reduce the dynamic response of the RC column structure, and play an effective protective role in the RC column. In these studies, although there are reference results of BHTS in RC beams, slabs, and composite columns, there is a lack of research on BHTS in RC columns, which cannot intuitively evaluate the protective effect of BHTS on RC columns. Therefore, in this paper, BHTS designed by Hao and Du [15] was used as an EA device for RCBP protection. Research was conducted on the failure mechanism of RC columns under varying levels of loading energy input, with an assessment of the effectiveness of BHTS in reducing damage to RCBP, which provides valuable guidance for engineers to protect RCBP from impact.

## 2. Experimental Methods and Material Numerical Models

### 2.1. Designing Experiments for RC Column

#### 2.1.1. Design and Manufacture of Model Pier

**Figure 1:**
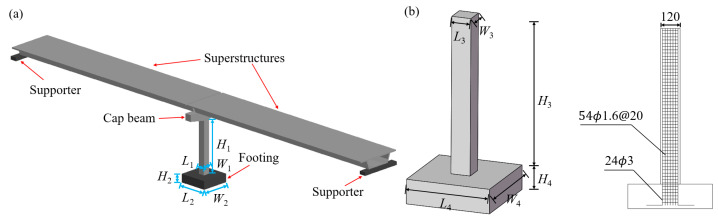
(**a**) Three-dimensional schematic of the bridge pier featuring the superstructures. (**b**) Bridge pier specimens.

The model in this study was designed based on the T-shaped single pier bridge investigated by Do et al. [21], as shown in Figure 1a. The cross section of the reference RCBP used in this research was 1200 mm × 1200 mm (*L*_1_ × *W*_1_), with a height of 9600 mm (*H*_1_). The concrete footing measured 5200 mm in length (*L*_2_), 5200 mm in width (*W*_2_), and 1500 mm in height (*H*_2_). The combined weight of the superstructure, cap beam, and column totaled approximately 4600 kN. According to reference [21], the pier was strengthened using 24 longitudinal steel reinforcements measuring 30 mm in diameter (*D*_1_), along with transverse steel reinforcements measuring 16 mm in diameter (*D*_2_) and spaced at intervals of 200 mm (@1). An RCBP with a scale ratio of 1:10 was designed by dimensional analysis [22,23] (Figure 1b). The model pier had a height of 960 mm, with dimensions of 120 mm for both width and length (*L*_3_ × *W*_3_ × *H*_3_). The column itself measured 520 mm in length, 520 mm in width, and 150 mm in height (*L*_4_ × *W*_4_ × *H*_4_). The model pier used longitudinal reinforcement made of iron wire with a diameter of 3 mm (*D*_3_), while the stirrup used iron wire with a diameter of 1.6 mm (*D*_4_) and a spacing of 20 mm (@2). The superstructure and the cap beam are transferred to the column by the 4.267 kN axial load applied by the jack.

#### 2.1.2. Test Scheme

**Figure 2:**
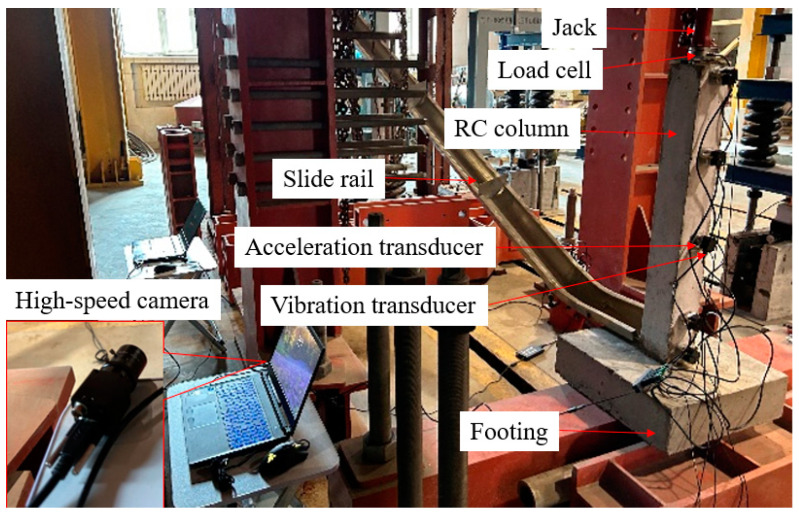
Horizontal collision device.

The RC column specimens were connected to the concrete foundation, and the total mass of each specimen was about 130 kg, as shown in Figure 2. The combination of the axial force and gravity ensured that the base had adequate horizontal friction and limited three-dimensional movement. A special device was created to simulate the lateral collision between a steel ball and a column, which includes a test steel ball and a slide rail. The steel ball used in the test has a radius of 62.5 mm and weighs a total of 8 kg. The angle between the slide rail and the horizontal ground is 25°. Before the test, a set of impact tests at a height of 1.5 m was carried out to calibrate the friction coefficient, take 0.15. Next, the height of impact was modified to match the desired impact energy by either extending or shortening the track. A load cell capable of handling up to 50 kN was placed on the top of the RC specimen column in order to measure the reaction force at the top of the column. The RC column on the iron plate measuring 120 mm × 120 mm × 10 mm uniformly received the reaction force. Acceleration transducers were placed at intervals of 0.05 m, 0.15 m, 0.42 m, 0.69 m, and 0.93 m from the foundation’s bottom to monitor the acceleration of the RC column. There are many different comparison conditions in the same batch of tests outside this study. One RC column and one BHTS-RC column were used in this impact test.

### 2.2. Materials

#### 2.2.1. Design and Layout of BHTS

**Figure 3:**
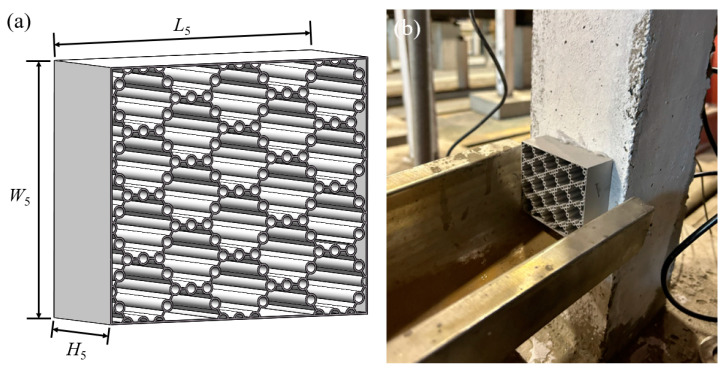
Impact test design: (**a**) BHTS geometry; (**b**) BHTS-RC column impact diagram.

The impact height of 0.15 m is the loading height of 1.5 m for the equivalent static region of the vehicle according to the AASHTO regulations of the United States Department of Transportation, formulated based on a 1:10 scale ratio. The length, width, and height of the BHTS buffer interlayer *L*_5_, *W*_5_, and *H*_5_ were 80 mm, 69.28 mm, and 30 mm, respectively. The fabricated BHTS was installed at a height of 150 mm vertically to the base, as shown in Figure 3. The material adopted the most commonly used AlSi10Mg in 3D printing, and the initial yield strength of the material is 220 ± 15 MPa. The material has excellent mechanical properties and is lightweight, which can ensure sufficient energy absorption. The details of the material can be seen in an independent study [16]. In addition, the fabrication process and material mechanical properties of BHTS have been described in detail in other independent studies [17,18,19,20].

#### 2.2.2. Materials Numerical Model

**Figure 4:**
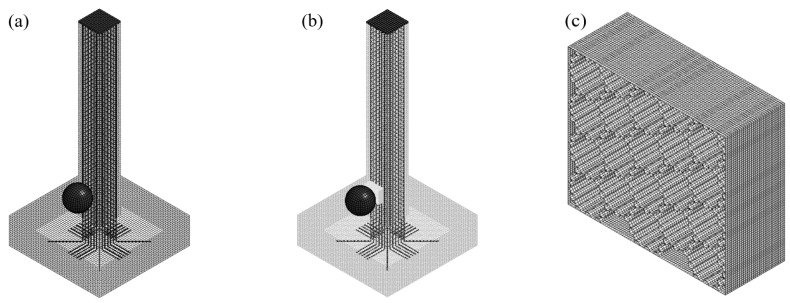
FEM of pier specimen: (**a**) RC column; (**b**) BHTS-RC column; (**c**) BHTS buffer interlayer.

In this study, the material model was used to verify the developed numerical model, and a three-dimensional nonlinear finite element model (FEM) of a steel ball impact RC column specimen test was established by finite element software LS-DYNA R11 [24,25], as shown in Figure 4a,b. The hexahedral solid element with reduced integral constant stress was used to model the concrete column, steel ball, and steel plate. The Hughes–Liu beam element with 2 × 2 Gauss integral was used to model the longitudinal reinforcement and stirrups in the simulation. The concrete adopted the *MAT_CONCRETE_DAMAGE_REL3 model commonly used in impact [24], and the state equation *EOS_TABULATED_COMPACTION [24,25] was chosen to accompany the aforementioned information.

In this model, the perfect bond between concrete and surrounding reinforcements was achieved by using *CONSTRAINED_LAGRANGE_IN_SOLID, as the bond-slip behavior of RC structure under low-velocity impact load can be neglected [26,27]. The single-sided contact algorithm *CONTACT_AUTOMATIC_SINGLE_SURFACE was used to contact the steel ball with BHTS and RC columns. The steel plate and concrete surface contact algorithm *CONTACT_AUTOMATIC_SURFACE_TO_SURFACE option was used [25,28]. In numerical simulations, gravity was applied using dynamic relaxation with the *LOAD_BODY_Z keyword, and stability was ensured with *CONTROL_DYNAMIC_RELAXATION. The *INITIAL_VELOCITY_GENERATION_START_TIME keyword was utilized to establish the starting point for velocity initialization. The BHTS structure was meshed using the fully integrated Belytschko–Tsay membrane element, as shown in Figure 4c. Three integration points were used along the thickness, with a mesh side length of 1 mm. The RC column specimen was contacted with BHTS using the binding contact algorithm *CONTACT_TIED_SHELL_EDGE_TO_SURFACE. Simultaneously, to avoid the intrusion of BHTS during compression self-contact, we opted for the single-sided automatic contact algorithm *CONTACT_AUTOMATIC_SINGLE_SURFACE. Three different mesh sizes, specifically 2.5 mm, 5 mm, and 10 mm, were examined. The 5 mm mesh showed satisfactory convergence, while the error compared to convergence for the 10 mm mesh was approximately 5%. Taking into account considerations such as grid sensitivity and computational efficiency [29], the 10 mm mesh was chosen as the optimal size for concrete due to its fast calculation speed and high accuracy.

#### 2.2.3. Model Verification

In the KCC model, the damage scalar is defined as the factor associated with the adjusted effective plastic strain, ranging from 0 to 2. Typically, a darker color indicates a higher level of damage according to previous research [18].

(1) Experimental phenomenon

**Figure 5:**
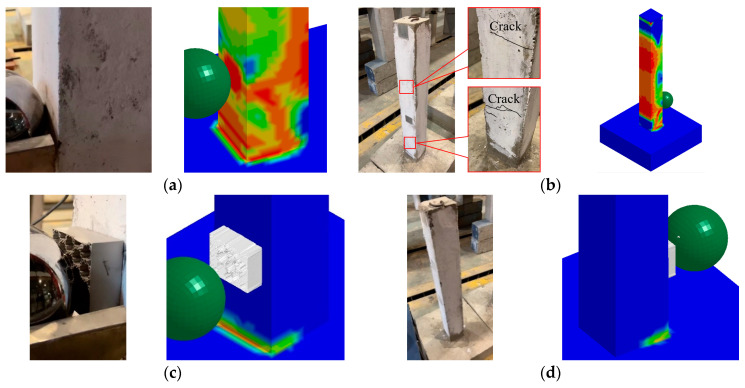
Comparison of failure phenomenon between test column and FEM: (**a**) impact front of RC column; (**b**) impact back of RC column; (**c**) impact front of BHTS-RC column; (**d**) impact back of BHTS-RC column.

During the test on the unprotected RC column, a faint white marker coating was observed to peel off at the impact site and its surrounding area, suggesting that the impact load from the steel ball had damaged the local concrete at the impact point (Figure 5a). Additionally, noticeable bending cracks emerged at the rear and upper side of the impact zone (Figure 5b). Bending cracks were also visible at approximately 650 mm above the base of the RC column, starting from the impact point. The distance between the back impact surface of the simulation results and the vertical position of the bottom was approximately 120–260 mm, and obvious bending damage marks appeared at 500–700 mm. There was no obvious damage to the BHTS-RC column (Figure 5c,d). There was no visible crack in the test, and the damage coefficient shown by the numerical cloud map was basically in the range of no damage [18]. In the experiment, the steel ball hit the upper position of BHTS, and obvious plastic deformation occurred. In the numerical simulation, the plastic deformation dominated by the same type of shear deformation as the experimental phenomenon occurred when the steel ball impacted the center of BHTS.

(2) Test results

**Figure 6:**
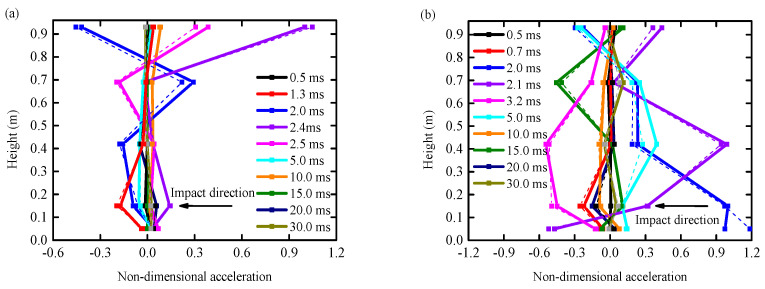
Comparison of test results: (**a**) RC column; (**b**) BHTS-RC column.

The test results for RC columns were analyzed in comparison to numerical simulation results, as shown in Figure 6. The average errors for columns with and without BHTS buffer interlayer protection were found to be 12.33% and 16.25%, respectively. The standard deviations for these results were 4.82% and 4.59%, respectively, meeting the criteria of less than 15% as specified in previous studies [30]. By comparing the experimental observations from the previous section, it is evident that the numerical analysis findings align well with the experimental data. The simulation effectively captures the dynamic response of the test column to impact loads. As a result, it is reasonable to conclude that the material model and associated numerical algorithm are suitable for analyzing the dynamic performance of similar structures under transverse impact loads.

## 3. Results

### 3.1. Impact Phenomenon

#### 3.1.1. Damage of RC Column

**Figure 7:**
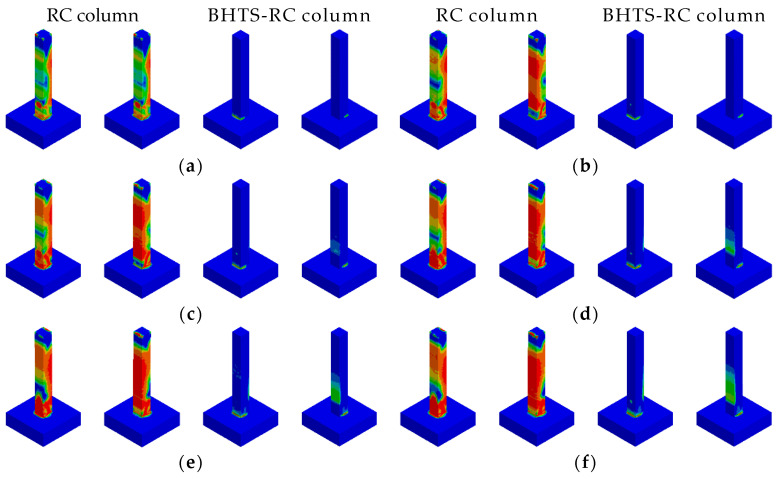
Comparison of failure phenomena of RC and BHTS-RC column under impact of steel balls at different heights: (**a**) 1.0 m; (**b**) 1.5 m; (**c**) 2.0 m; (**d**) 2.5 m; (**e**) 3.0 m; (**f**) 3.5 m.

The BHTS buffer interlayer device was positioned 0.15 m above the base of the pier. Numerical simulation tests were conducted on the RC column to assess energy impact at equivalent heights of 1.0, 1.5, 2.0, 2.5, 3.0, and 3.5 m, respectively. The results showed that the RC column was seriously damaged on the back of the impact position, and there was also no obvious shear damage. There was no obvious damage to the RC column at the impact height of 1.0 m (Figure 7a). With the increase in the input energy, there were obvious diagonal cracks in the RC column at the impact height of 1.5 m, and there were also obvious transverse cracks on the back of the impact (Figure 7b). At the impact height of 2.0–3.5 m, with the increased impact energy, the cracking range of concrete on the back of the impact was further increased (Figure 7c–f). The damage of BHTS-RC column under all loading conditions (1.0–3.5 m) was quite different from that of the RC column (Figure 7a–f), and there was no obvious damage phenomenon in the RC column. Similar to the RC column, the plastic deformation of the BHTS-RC column was more obvious with the increase in the loading energy. One possible explanation is that the RC column experienced significant base shear force and inertial force when subjected to a pulse load, leading to a tendency towards shear failure. However, by incorporating a BHTS buffer interlayer, the base shear force and inertia force acting on the RC column were reduced during impact loading, causing the BHTS-RC column to lean towards a bending failure mode.

#### 3.1.2. BHTS Damage Situation

**Figure 8:**
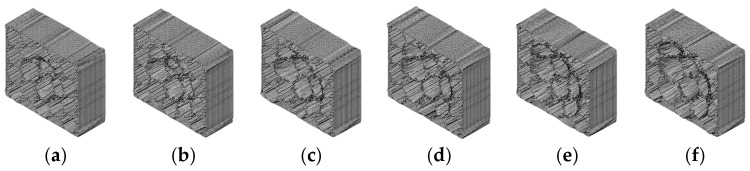
Deformation of BHTS at different impact heights: (**a**) 1.0 m; (**b**) 1.5 m; (**c**) 2.0 m; (**d**) 2.5 m; (**e**) 3.0 m; (**f**) 3.5 m.

Figure 8 illustrates that as the impact height increases, the damage degree of BHTS also increases. The three primary deformation modes of BHTS include linear and nonlinear elastic deformation, plastic deformation from shear and crushing, and local flexural buckling. Plastic deformation is the main way for BHTS to absorb impact kinetic energy.

### 3.2. Impact Force Comparison

**Figure 9:**
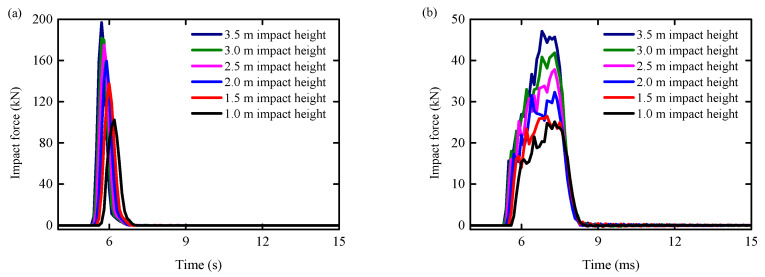
Time history curve of impact force: (**a**) RC column; (**b**) BHTS-RC column.

Figure 9 displays the time history curve of impact force for each set of specimens. It is evident that the impact force on all reinforced concrete columns rises in tandem with the height at which the steel ball is dropped. The average increase in impact force between the 1.5–3.5 m and the 1.0 m impact condition in the RC column condition is 43.76%. For the BHTS-RC column, the impact force experienced an average increase of 33.75% when subjected to impact conditions ranging from 1.5 m to 3.5 m, compared to the impact force under the 1.0 m condition.

**Figure 10:**
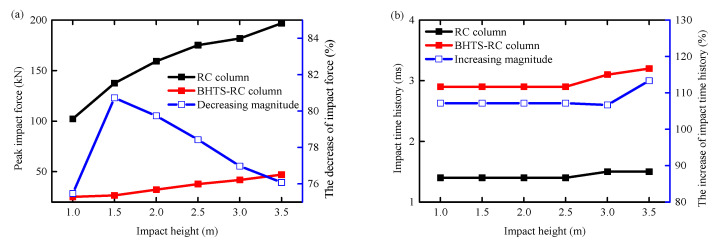
Comparison of impact force and impact time history: (**a**) impact force; (**b**) impact time history.

A comparison of impact force and impact time history between unprotected and BHTS-protected RC columns is presented in Figure 10, illustrating the relationship between the two. The results demonstrate that the BHTS buffer interlayer effectively mitigates the impact force on RC columns, with an average reduction of 77.89%. Additionally, the BHTS buffer interlayer extends the impact time by an average of 108.09%, thereby reducing the inertia force significantly.

### 3.3. Displacement Comparison

**Figure 11:**
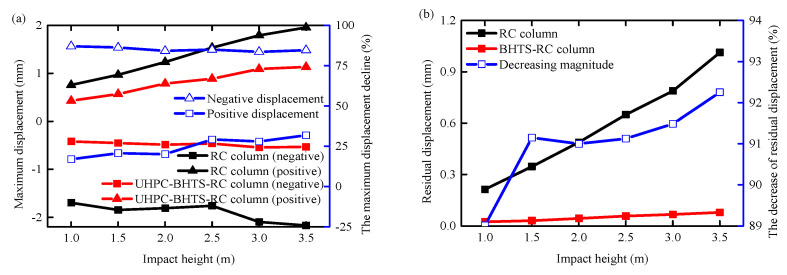
Comparison of maximum and residual displacement: (**a**) maximum displacement; (**b**) residual displacement.

Figure 11 illustrates the maximum lateral and residual displacement of RC columns under different operating conditions. Comparing the RC column with a BHTS buffer interlayer to one without any protective device, it is evident that the former exhibits smaller maximum and residual displacement. The data clearly indicate that the BHTS buffer interlayer plays a crucial role in reducing both displacement response and residual displacement of the RC column. On average, the maximum positive/negative displacement decreased by 24.47%/85.11%, while the residual displacement is reduced by 91.00%.

## 4. The Protective Effect and Failure Mode of BHTS on RC Columns

At present, many researchers have extensively studied the impact protection of beams, plates, and columns [31,32,33,34,35]. The failure modes of RC structures mainly include shear failure and bending failure [36,37,38,39]. To facilitate the study of material damage and failure conversion modes in RC columns with or without protective devices under shear and bending failure, a 20 m equivalent height impact scenario involving a high-energy steel ball was devised.

### 4.1. Failure Phenomenon

#### 4.1.1. RC Column

**Figure 12:**
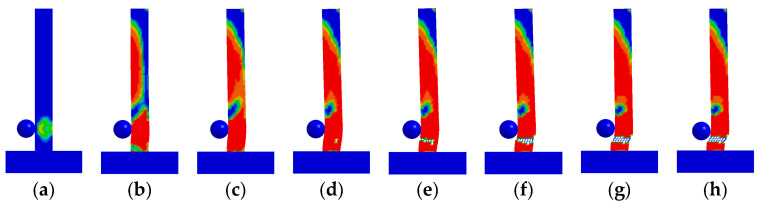
Development process of RC column concrete damage: (**a**) 5.3 ms; (**b**) 6.0 ms; (**c**) 7.0 ms; (**d**) 8.1 ms; (**e**) 8.5 ms; (**f**) 12 ms; (**g**) 35 ms; (**h**) 100 ms.

Figure 12 shows the concrete damage development process of unprotected RC columns. The impact initiation time (the starting time of the abscissa) is 5.2 ms. When *t* ≤ 6.0 ms, the concrete at the back of the impact position and the bottom and upper part of the impact surface first appeared tensile damage, and cracks appeared successively at each position. When 7.0 ms ≤ *t* ≤ 8.1 ms, a principal diagonal crack appeared at the bottom of the collision surface and gradually developed downward to a position about 5 cm from the bottom of the back collision surface, thus forming a penetrating diagonal crack. When 8.1 ms ≤ *t* ≤ 100 ms, the damage degree of the principal diagonal crack gradually increases, and the damage factor reaches the damage level. The strain of the concrete element at the crack position was deleted because it reached the set threshold and lost its bearing capacity. The RC column underwent shear (punch) failure under the combined action of impact force and axial force.

#### 4.1.2. BHTS-RC Column

**Figure 13:**
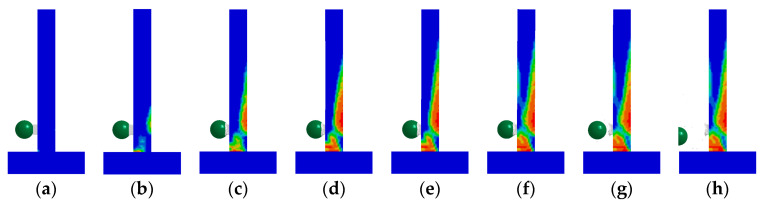
Development process of BHTS-RC column concrete damage: (**a**) 5.4 ms; (**b**) 6.0 ms; (**c**) 7.0 ms; (**d**) 8.0 ms; (**e**) 8.5 ms; (**f**) 12 ms; (**g**) 35 ms; (**h**) 100 ms.

Figure 13 shows the concrete failure development process of BHTS-RC column. It can be seen from the diagram that the bending damage of the RC column is located at the bottom section of the column and the back of the impacted point. When *t* ≤ 7.0 ms, slight tensile damage occurred at the back of the impact point and the bottom of the impact surface. The area of the compression zone at the bottom of the column section decreased rapidly, and the height of the compression zone accounts for about 30% of the height of the section. When 7.0 ms < *t* ≤ 100 ms, the concrete damage around the longitudinal reinforcement increased rapidly, and the area of the compression zone at the bottom section of the column decreased continuously. However, the damage at the rear of the point of impact gradually manifested over time. The BHTS-RC column experienced bending damage due to both the impact force and axial force acting together.

### 4.2. Analysis of Failure Mechanism

**Figure 14:**
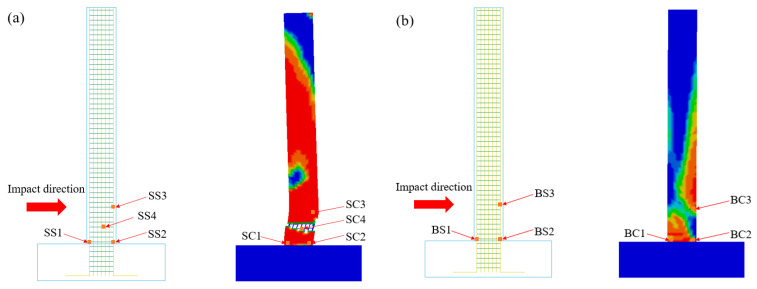
Development process of RC column concrete damage: (**a**) position diagram of shear failure observation unit; (**b**) position diagram of bending failure observation unit.

In order to facilitate the analysis and description, Figure 14a shows the location of the shear failure related feature units. Among them, SS1 and SS2 are the longitudinal main reinforcement elements at the bottom of the column. SS3 is the longitudinal main reinforcement unit on the back of the impact point. SS4 is a stirrup element at the position of the shear diagonal crack. SC1–SC4 are concrete elements at the corresponding positions of reinforcement elements SS1–SS4, respectively. In order to facilitate the analysis and description, Figure 14b shows the position diagram of the characteristic elements related to bending failure. BS1 and BS2 are the longitudinal main reinforcement elements at the bottom of the column. BS3 is the longitudinal main reinforcement element on the back of the impact point. BC1, BC2, and BC3 are concrete elements at the corresponding positions of BS1, BS2, and BS3, respectively.

#### 4.2.1. Damage Behavior of Concrete

**Figure 15:**
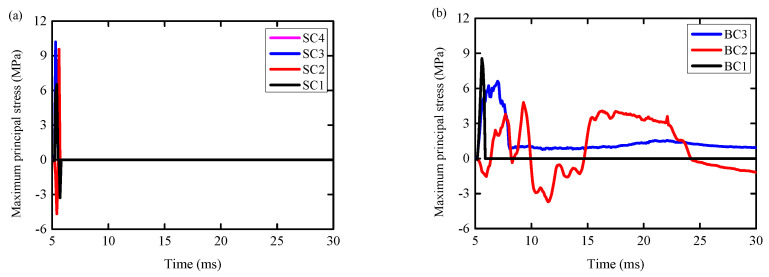
Maximum principal stress of shear and bending concrete elements: (**a**) RC column; (**b**) BHTS-RC column.

**Figure 16:**
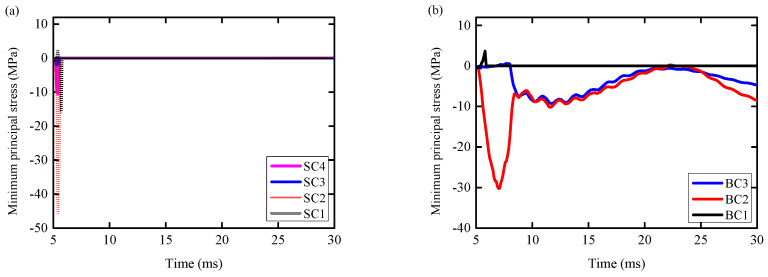
Minimum principal stresses of shear and flexural concrete elements: (**a**) RC columns; (**b**) BHTS-RC column.

(1) It can be seen from Figure 15a that the maximum principal stress of the concrete elements SC1 and SC3 on the RC column reached the peak at 5.4 ms (6.52 MPa/10.18 MPa). At the same time, the value of the maximum principal stress decreased to zero at 5.5 ms, and the element was completely destroyed. In this process, the minimum principal stress of concrete elements SC1 and SC3 (−2.12 MPa/−2.07 MPa) was much smaller than the compressive strength of concrete (Figure 16a), indicating that the tensile failure of concrete at SC1 and SC3 occurred. The maximum principal stresses of concrete elements SC2 and SC4 reached the peak values (9.55 MPa and 7.79 MPa) at 5.6 ms and 5.4 ms, respectively, and the values of the maximum principal stresses decreased to zero at 5.6 ms and 5.4 ms, respectively. From Figure 15b, it can be seen that the maximum principal stresses of BC1 and BC3 concrete elements on RC columns under BHTS-RC column conditions reached the peak values (8.56 MPa/6.61 MPa) at 5.6 ms and 7.0 ms, respectively. The maximum principal stress of BC1 decreased to zero at 5.9 ms, and the element was completely destroyed. At 8.1 ms, the maximum principal stress of BC3 decreased to 0.79 MPa, and the unit was severely damaged. At the same time, the minimum principal stress of concrete elements BC1 and BC3 (3.64 MPa/−9.25 MPa) was much smaller than the compressive strength of concrete (Figure 16b), indicating that the tensile failure of concrete at BC1 and BC3 occurred. The maximum principal stress of concrete element BC2 reached the peak value (4.80 MPa) at 9.3 ms and decreased to zero at 9.9 ms. At this time, the minimum principal stress of the concrete unit BC2 was −8.01 MPa, which did not reach the compressive strength of the concrete, indicating that the main factor for the formation of concrete cracks here was tensile stress.
(2) It can be seen from Figure 16a that the minimum principal stress (−45.55 MPa) of the concrete element SC2 of the RC column reached the compressive strength of the concrete, indicating that the place was controlled by both tensile and compressive stresses. The minimum principal stress of SC4 (−10.54 MPa) was far from the compressive strength of concrete, indicating that the formation of concrete diagonal cracks was mainly controlled by the maximum principal stress (tensile stress). At the same time, the minimum principal stress (3.64 MPa/−9.25 MPa) of BC1 and BC3 concrete units of BHTS-RC column is much smaller than the compressive strength of concrete (Figure 16b), indicating that the tensile failure of concrete at BC1 and BC3 occurred.

#### 4.2.2. Damage Behavior of Reinforcement

**Figure 17:**
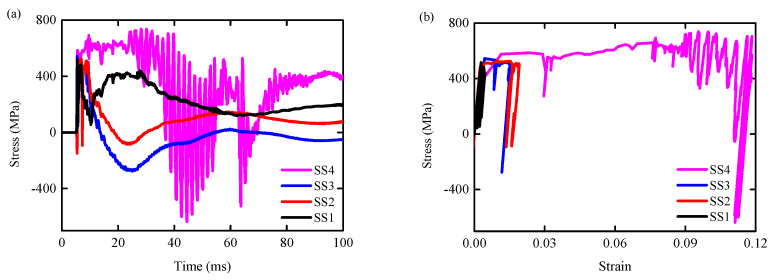
Axial stress time history curve of steel reinforcement element SS1–SS4/BS1–BS3: (**a**) RC column; (**b**) BHTS-RC column.

Figure 17a,b are the axial stress time history curves and stress-strain time history curves of the steel reinforcement elements SS1–SS4, respectively. It can be seen from the figures that the steel reinforcement elements SS1–SS4 had yielded, meaning that the plastic hinge had been formed at the bottom of the column. The yield stresses are 514 MPa, 485 MPa, 543 MPa, and 575 MPa, respectively.

**Figure 18:**
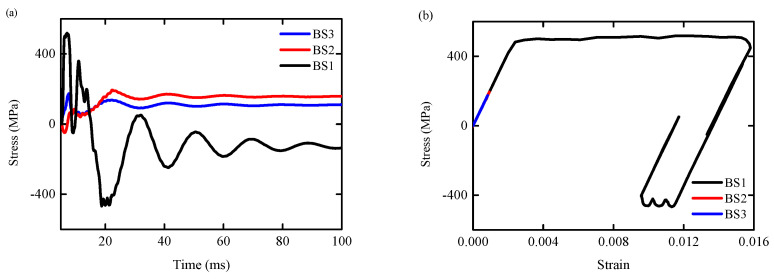
Axial stress-strain curve of reinforcement element SS1–SS4/BS1–BS3: (**a**) RC column; (**b**) BHTS-RC column.

Figure 18a,b show the axial stress time history curve and stress-strain time history curve of the reinforcement elements BS1–BS3, respectively. It can be seen from the diagrams that before the failure of the RC column, the reinforcement element BS1 had yielded, meaning that a plastic hinge had formed in the tensile area at the bottom of the column, with a yield stress of 482 MPa. The maximum stress for BS2 and BS3 reinforcement elements is 194 MPa and 173 MPa, respectively.

### 4.3. Impact Force

**Figure 19:**
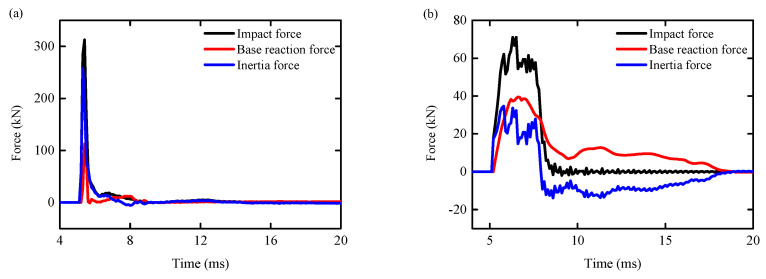
Dynamic time history curve: (**a**) RC column; (**b**) BHTS-RC column.

Figure 19a,b display the time history curves for the impact force, the base horizontal reaction force, and the RC column inertia force. Comparing the shear failure in Figure 19a with the bending failure in Figure 19b, the bending-shear failure of the RC column exhibits a similar impact force time history curve. The data clearly shows that the inertia force and base reaction force were more pronounced in cases of shear failure in the RC columns without the BHTS anti-collision device. On the other hand, the impact force, base reaction force, and inertia force were all lower in the RC column with BHTS. In comparison with the RC column without a protective device, they are 77.24%, 65.17%, and 86.77% smaller, respectively, and the peak impact time is extended by 183.33%, suggesting that the anti-collision device successfully shielded the RC column.

### 4.4. Horizontal Displacement

**Figure 20:**
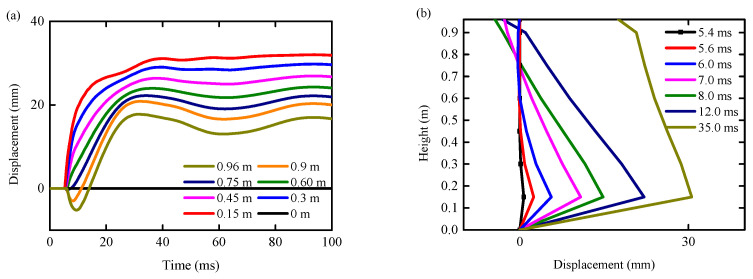
Horizontal displacement curves of each height under shear condition: (**a**) time history curve; (**b**) distribution curve with respect to height.

Figure 20 shows the horizontal displacement time history curve of each observation point under the condition of an RC column and the horizontal displacement curve of the central axis of an RC column at a typical time. When *t* < 9 ms, the RC column began to undergo large local shear deformation near the impact point, and then the overall bending deformation occurred. When 9 ms < *t* < 100 ms, the local shear deformation near the bottom of the column increased slowly, and the bending deformation of the RC column also increased slowly until the RC column was completely sheared. Throughout the impact process, the deformation pattern of the RC column underwent a rapid transition. Initially, it started with local shear deformation at the point of impact then shifted to overall bending deformation and ultimately culminated in shear failure primarily driven by local shear deformation at the impact site. This demonstrates that the deformation of the unprotected RC column during the impact process can be divided into two modes: local shear deformation and overall bending deformation. The main reason for the increase in overall bending deformation is the formation of plastic hinges at the bottom of the column.

**Figure 21:**
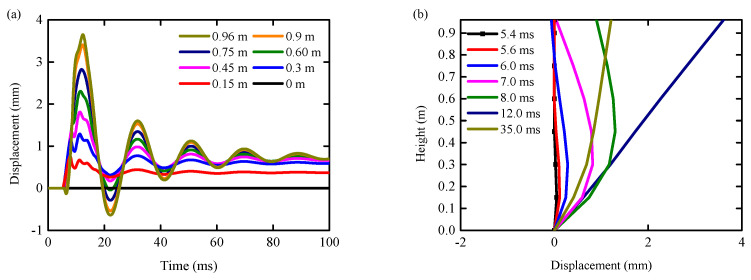
Horizontal displacement curves of each height under bending condition: (**a**) time history curve; (**b**) distribution curve with respect to height.

The diagram in Figure 21 illustrates the horizontal displacement over time for different observation points under the BHTS-RC column condition, as well as the horizontal displacement curve for the central axis of the RC column at a specific moment. The diagram reveals that there are two types of bending deformation exhibited by RC columns during the impact process: local bending deformation and overall bending deformation. In the initial stage of the impact (*t* < 8 ms), the RC column was dominated by local bending deformation, and the maximum deformation position was near the impact point. After that, the deformation model of the RC column was dominated by overall bending deformation. The displacement of the top of the RC column gradually increased and began to be greater than the horizontal displacement at the impact point. When *t* > 40 ms, the overall bending deformation of the RC column was basically restored.

### 4.5. Energy Conversion

**Figure 22:**
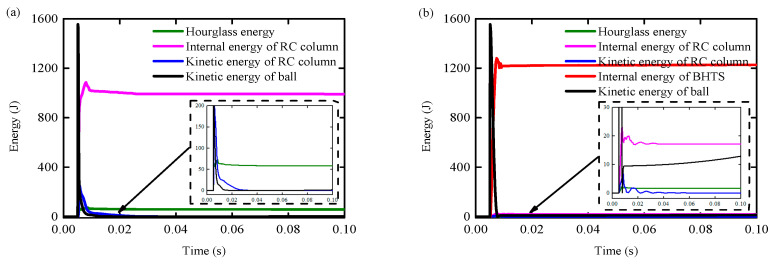
Time history curve of energy change: (**a**) RC column; (**b**) BHTS-RC column.

Figure 22 illustrates the variation of energy throughout the impact process under both RC column and BHTS-RC conditions. Some components of energy, such as hourglass energy, sliding energy, kinetic energy, and internal energy, do not fully account for the overall energy consumption. This observation suggests that:

(1) Quasi-elastic stage (I): In the RC condition, *t* ≤ 5.2 ms, the kinetic energy loss of the steel ball accounted for about 1.1% of the total impact kinetic energy. Among them, the RC column was the main EA factor, and the internal energy accounts for about 40.90% of the energy loss at this stage. In the BHTS-RC condition, *t* ≤ 5.9 ms, the kinetic energy loss of the steel ball accounted for about 34.54% of the total impact kinetic energy. Among them, BHTS was the main EA factor, and the internal energy accounted for about 87.89% of the energy loss at this stage.
(2) Working stage with cracks (II): In the RC condition, 5.2 < *t* ≤ 5.3 ms, the kinetic energy loss of the steel ball accounted for about 21.78% of the total impact kinetic energy. Among them, the RC column was the main EA factor, and the internal energy accounted for about 61.07% of the energy loss at this stage. In the BHTS-RC condition, 5.9 ms < *t* ≤ 6.1 ms, the kinetic energy loss of the steel ball accounted for about 12.63% of the total impact kinetic energy. Among them, BHTS was the main EA factor, and the internal energy accounted for about 89.83% of the energy loss at this stage.
(3) Yield stage (III): In the RC condition, 5.3 ms < *t* ≤ 5.4 ms, the kinetic energy loss of the steel ball accounted for about 34.46% of the total impact kinetic energy. Among them, the RC column was the main EA factor, and the internal energy accounted for about 67.31% of the energy loss at this stage. In the BHTS-RC condition, 6.1 ms < *t* ≤ 6.6 ms, the kinetic energy loss of the steel ball accounted for about 26.67% of the total impact kinetic energy. Among them, BHTS was the main EA factor, and the internal energy accounted for about 82.97% of the energy loss at this stage.
(4) Failure stage (IV): In the RC condition, *t* > 5.4 ms, the kinetic energy loss of the steel ball accounted for about 42.45% of the total impact kinetic energy. Among them, the RC column was the main EA factor, and the internal energy accounted for about 77.28% of the energy loss at this stage. In the BHTS-RC condition, *t* > 6.6 ms, the kinetic energy loss of the steel ball accounted for about 25.32% of the total impact kinetic energy. Among them, BHTS was the main EA factor, and the internal energy accounted for about 73.04% of the energy loss at this stage.

## 5. Conclusions

This study thoroughly investigated the protective capabilities of a bio-inspired honeycomb column thin-walled structure on an RC column subjected to impact loading through a combination of experiments and numerical simulations. By analyzing the effects on impact force, displacement, energy dissipation mechanisms, and failure modes of RC columns, the following conclusions were drawn:

1. The presence of the BHTS buffer interlayer resulted in a decrease in the impact force experienced by the RC column and also extended the duration of the impact. On average, the impact force on the BHTS-RC column was 77.89% lower compared to the RC column, leading to a significant reduction in impact force. Additionally, the use of BHTS prolonged the impact time history by an average of 108.09%, effectively reducing the inertial force.
2. The use of a BHTS buffer interlayer has been shown to greatly decrease the displacement response and residual displacement of reinforced concrete columns. On average, the maximum positive/negative displacement of the RC column was reduced by 24.47%/85.11%, while the residual displacement saw an average reduction of 91.00%.
3. During the different stages of deformation, including the quasi-elastic stage, the working stage with cracks, the yield stage, and the failure stage, the RC column played a crucial role in absorbing energy. Specifically, the concrete within the RC column absorbed 69.76% of the kinetic energy from the steel ball. On the other hand, in the BHTS-RC condition, the BHTS absorbed 82.33% of the kinetic energy from the steel ball, demonstrating its effectiveness in absorbing energy.
4. Through an analysis of material damage, impact force, displacement, and energy absorption characteristics during the failure stage, it was found that the BHTS buffer interlayer was able to transform the failure mode of the RC column from shear failure to a less severe bending failure.

Studies have shown that BHTS has excellent energy absorption performance and can form an effective protective effect on RC columns under impact. However, BHTS is prone to local plastic deformation during use, which affects its efficiency and durability. It is necessary to increase the protective structure as a panel layer to improve its overall performance. Therefore, in future research, BHTS can be combined with some excellent ductile materials to form a combined structure for impact protection engineering.

## Data Availability

The raw data supporting the conclusions of this article will be made available by the authors on request.
